# From Omic Layers to Personalized Medicine in Colorectal Cancer: The Road Ahead

**DOI:** 10.3390/genes14071430

**Published:** 2023-07-11

**Authors:** Irati Romero-Garmendia, Koldo Garcia-Etxebarria

**Affiliations:** 1 Department of Genetics, Physical Anthropology and Animal Physiology, University of the Basque Country (Universidad del País Vasco/Euskal Herriko Unibertsitatea), 48940 Leioa, Spain; irati.romero@ehu.eus; 2 Biodonostia, Gastrointestinal Genetics Group, 20014 San Sebastián, Spain; 3 Centro de Investigación Biomédica en Red de Enfermedades Hepáticas y Digestivas (CIBERehd), 08036 Barcelona, Spain

**Keywords:** colorectal cancer, genetics, genomics, transcriptomics, microbiota, personalized medicine

## Abstract

Colorectal cancer is a major health concern since it is a highly diagnosed cancer and the second cause of death among cancers. Thus, the most suitable biomarkers for its diagnosis, prognosis, and treatment have been studied to improve and personalize the prevention and clinical management of colorectal cancer. The emergence of omic techniques has provided a great opportunity to better study CRC and make personalized medicine feasible. In this review, we will try to summarize how the analysis of the omic layers can be useful for personalized medicine and the existing difficulties. We will discuss how single and multiple omic layer analyses have been used to improve the prediction of the risk of CRC and its outcomes and how to overcome the challenges in the use of omic layers in personalized medicine.

## 1. Introduction

Worldwide, approximately 10% of diagnosed cancers are colorectal cancer (CRC), and it is the second cause of death among cancers [1,2]. Moreover, 500,000 cases of CRC are diagnosed and 242,000 persons die as a consequence of CRC each year in Europe alone [3]. CRC affects both sexes and European countries similarly: In females, it is the second most diagnosed cancer and the third cause of death, and in males, it is the third most diagnosed cancer and the second cause, and the majority of European countries show this trend [3]. All of these numbers suggest that CRC has a high burden in developed countries and is a major concern for health systems. To address the burden that is CRC, screening strategies have been developed to facilitate its detection, and the search for biomarkers for more accurate diagnosis, prognosis, and success of treatment of CRC is in constant development [4]. There is no doubt that the better known the biological factors that contribute to the risk and etiology of CRC are, the better the biomarkers that will be found.

CRC shows a great heterogeneity since the majority of CRC cases are sporadic and the minority are genetically inherited. Lynch syndrome (2–4% of diagnosed CRCs) and polyposis syndromes (e.g., adenomatous polyps, Peutz–Jeghers polyps, or serrated polyps) are the two main inherited syndromes [5]. Hypermutated cancers with microsatellite instability and non-hypermutated cancers with copy number alterations are the main classes of sporadic tumors [6]. In addition, sporadic CRCs are developed in two main ways [7]: In approximately 66% of sporadic CRC, conventional adenomas (lesions with tubular, tubulovillous, or villous histology) can progress to CRC (the conventional way); in the rest of the cases, serrated adenomas (lesions with the stellate architecture of the crypt epithelium) can progress to CRC (serrated way).

Lifestyle and the environment are among the risk factors for CRC, since its development is influenced by diet and physical activity [8]. In addition, the previous diseases suffered by patients are another source of risk, since inflammatory processes can lead to the development of CRC [9,10], especially in the case of inflammatory bowel disease [11].

Although “personalized medicine” has been used with different meanings, it can be defined as the right treatment for the right person at the right time [12]. The development of omic technologies has made possible the identification of individual susceptibility to some diseases and the response to therapies and, therefore, better prediction, prevention, diagnosis, and treatment of diseases could be reached [12]. In cancer, the use of personalized medicine approaches can be used for screening persons with a higher risk of developing the disease using genetic tests that can identify risks or determine the best treatment for a patient based on the molecular characterization of cancer to foresee the disease progression and the response to the treatment [12].

In this review, we will try to summarize how the analysis of the omic layers can be useful for personalized medicine and existing difficulties. First, we will briefly summarize the analysis of a single omic layer carried out in CRC; then, we will review the analyses carried out combining more than one omic layer, and we will finish with the conclusions and the possible steps to overcome the difficulties that we are facing when we want to implement personalized medicine in CRC.

## 2. Single Omic Layer Analyses

Since it is a topic of high interest, the available omic technologies or analyses and their contribution to CRC studies have been extensively reviewed before [13,14]. The focus of this review is not to re-summarize what information each omic layer can offer; therefore, in this section, we will try to summarize what information from each omic layer has been used towards personalized medicine.

### 2.1. Genome

Genome-wide association studies (GWASs) have been used to find the single nucleotide polymorphisms (SNPs) involved in the risk of developing CRC. In a meta-analysis of 16 studies, 34,626 CRC cases and 71,379 were analyzed and 623 SNPs from 79 loci were significantly associated with CRC risk in a population with European ancestry [15]. Based on some of those GWASs and additional GWAS analyses that have been carried out in recent years, polygenic risk scores (PRSs) have been developed to calculate the risk of an individual developing CRC [16,17,18,19,20,21,22,23,24]. However, the performance of the PRS is dependent on each model and the population in which it is applied [25]. Thus, researchers should evaluate what is the most appropriate PRS model to perform an optimal risk stratification in a given population, both in populations of the same ancestry and different ancestries [25,26].

Moreover, it has been observed that the SNPs involved in the risk of right colon cancer and left colon cancer are partly different [27]. The stratification of the analyses is the only way to detect the effect of some SNPs that could be involved in the development of CRC since they seem to be associated with the location of the tumor [25,27]. Thus, risk-stratification strategies should incorporate the differences in the genetic architecture according to the location and personalize accordingly the screenings.

GWASs have been carried out to find SNPs associated with metastatic colorectal cancer survival in treatment with chemotherapy plus biologics [28], survival in rectal cancer [29], progression-free survival in metastatic colorectal cancer in different treatments [30], and survival in colorectal cancer [31]. Thus, there are available markers that could be helpful to predict the success of the treatment and tailor the clinical options, although the validation of those markers in different populations is pending.

### 2.2. Epigenome

DNA methylation, histone modifications, and non-coding RNAs have been analyzed in CRC to find feasible biomarkers for different features of CRC.

The CpG island methylator phenotype, where the promoters of several genes (e.g., *hMLH1*, *CDKN2A*, *MINT*, *CACNA1G*, *CRABP1*, *IGF2*, *NEUROG1*, *RUNX3*, and *SOCS1*) show higher hypermethylation, has been proposed as a useful predictor of CRC [32,33,34]. In addition, the methylation status of *SEPT9* has been proposed for CRC diagnosis, although its performance is variable depending on the stage [35].

In the case of histone modifications, several modifications have been reported, although the most relevant modifications in CRC were acetylation and methylation [36,37]. In addition, H4K12ac and H3K18ac modifications increased in CRC [37,38].

Finally, the role of micro RNAs (miRNAs) and long non-coding RNAs (lncRNAs) in CRC could be relevant since their expression could affect oncogenic genes [39]. The expression of miRNAs was useful to distinguish between subgroups [40,41] and they have been proposed to help in finding new therapeutic targets [42]. In addition, the expression of lncRNAs has been used to distinguish between stages and progression [43,44,45].

It has to be highlighted that based on epigenetic markers, several biomarkers have been commercialized and proposed for clinical use or guidelines [46]. Thus, the epigenome seems to be an appropriate layer to find useful biomarkers.

### 2.3. Transcriptome

Transcriptome data were used to define the consensus molecular subtypes of CRC, a classification that is widely used [47]. Based on the gene-expression profiles of several transcriptome studies, four subtypes were defined [47]: CMS1, which showed microsatellite instability and immune filtration and activation; CMS2, which was defined as canonical; CMS3, which showed metabolic deregulation; and CMS4, which showed stromal infiltration. Recently, the use of single-cell transcriptome analysis was used to refine this classification, the intrinsic subtypes [48]. Two intrinsic subtypes were defined based on the transcriptome of epithelial cells [48]: iCMS2, which showed a mutation in *APC* and *TP53* and greater Wnt/b-catenin and *MYC* activity; and iCMS3, which showed KRAS, PIK3CA, and BRAM mutations and higher inflammation response.

Moreover, the transcriptome data of CRC available through TCGA [49] has been reanalyzed to find relevant genes related to different features of CRC. A differentially expressed gene analysis of CRC stages found 11 genes (*NEK4*, *RNF34*, *HIST3H2BB*, *NUDT6*, *LRCh4*, *GLB1L*, *HIST2H4A*, *TMEM79*, *AMIGO2*, *C20orf135,* and *SPSB3*) that change their expression depending on the stage [50]. For example, the *NEK4* gene, which is involved in the senescence of cells, showed higher expression in stage I and the lowest expression in stage IV [50]. However, the whole gene expression pattern of the CRC patients was not different between stages since the principal component analysis was not able to show clear patterns [50]. In addition, TCGA data were used to find genes associated with overall survival [51]. It was concluded that the expression pattern of six genes (*ART5*, *FOXD1*, *HIST3H2BB*, *TIMP1*, *EPHA6,* and *IRX6*) was able to discriminate between CRC patients with poor diagnostic outcomes and good diagnostic outcomes and that the model was independent of other clinical features [51].

Furthermore, the role of genes involved in DNA damage and repair mechanisms in colon cancer was interrogated [52]. First, part of the TCGA expression data was used to build a 12-gene model (*CCNB3*, *ISY1*, *CDC25C*, *SMC1B*, *MC1R*, *LSP1P4*, *RIN2*, *TPM1*, *ELL3*, *POLG*, *CD36*, and *NEK4*) that was able to differentiate low-risk and high-risk groups [52]. Then, the 12-gene model was applied to the remaining TCGA expression data, and the ability to classify 5 years of survival reached an area under the curve (AUC) of the receiver operating characteristic (ROC) of 0.79 [52]. In addition, the model was applied to two datasets available in the GEO database [52]; in one of the datasets, the AUC for survival of 5 years was 0.65, and in the other, it was 0.72.

Considering that the overlap of genes between studies is limited and the performance of the models is not ideal, it could be difficult to translate the results of the transcriptome into clinical practice.

### 2.4. Proteome

Proteome analysis has been used in different tissues (CRC biopsies, paraffin-embedded CRC biopsies, serum, and plasma) to find diagnostic biomarkers using several methods.

For example, a study where two-dimensional gel electrophoresis coupled to mass spectrometry was used on CRC biopsies and adjacent normal tissue found the upregulation of ACTBL2 [53]. In another study, Fourier transform mass spectrometry was used to analyze CRC biopsies and adjacent normal tissue, and the upregulation of DPEP1 was found [54]. Using liquid chromatography-mass tandem mass spectrometry on paraffine-embedded tissues, a study found that OLFM4, KNG1, and Sec24C have differential expression in the early CRC stages than in normal and premalignant tissues [55]. In another study, using the same method, CyPA, ANXA2, and ALDOA were found to be upregulated in CRC [56].

Moreover, another study, in which targeted liquid chromatography-tandem mass spectrometry was used on blood samples, proposed a model based on LRG1, EGFR, ITIH4, Hpx, and SOD3 proteins that had a good performance for CRC detection [57]. Another study, where liquid chromatography/multiple-reaction monitoring-mass spectrometry was used on plasma samples, proposed a different model for CRC detection based on MASP-1, SPP1, PON3, TfR1, and AREG [58]. In a study where matrix-assisted laser-desorption/ionization time-of-flight was used on serum samples, it was detected that the downregulation of STK4 was a good predictor for CRC diagnosis and possibly for distant metastasis [59]. In addition, the combination of high-performance liquid chromatography and mass spectrometry on serum samples found that MRC1 and S100A9 were upregulated in CRC [60].

As happened with the transcriptome, the overlap of proteins between studies is limited, and, therefore, their use for personalized medicine could face difficulties.

### 2.5. Metabolome

Several studies have analyzed the metabolome to find feasible candidates for CRC progression detection.

For example, fecal samples of CRC, adenoma, and healthy controls were examined to find metabolites that could differentiate between the three statuses [61]. In total, 105 metabolites were evaluated, and 18 of them were altered in CRC [61]. In addition, a predictive model was constructed using seven metabolites, and the AUC of the model was 0.821 [61]. The inclusion of sex and age improved the model (AUC = 0.848) and the results of the fecal occult blood test (AUC = 0.885) [61]. In addition, 1380 metabolites were analyzed in CRC, adenoma, and healthy controls, and 25 metabolites were found to differentiate CRC and adenomas from healthy controls. Among those metabolites, sphingomyelins, lactosylceramides, and secondary bile acids were detected [62]. The combination of five metabolites showed an accuracy of 91.67% [62].

Moreover, 50 lipids were found to be good biomarkers for the adenoma-to-CRC sequence, especially phosphatidylcholines and triacylglycerols [63]. The use of four metabolites showed a good performance in differentiating the different statuses (adenoma from normal, AUC = 0.879; CRC from normal, AUC = 0.817; CRC from adenoma, AUC = 0.805) [63]. In another study, 79 lipids were found to have differential abundance between CRC and controls, the majority of which were phosphatidylcholines and triacylglycerols [64]. From those lipids, 12 lipids showed an AUC > 0.95 [64].

Considering the performance of the models, metabolites seem to be feasible biomarkers for CRC, although the detected metabolites could not be the same in different studies.

### 2.6. Microbiome

Microbiome has been extensively investigated to find markers that could be useful to predict the development of CRC, especially in fecal samples since it is the less invasive method [65]. In CRC, some taxa are different compared with healthy samples, although there is variability among studies [66,67,68]. In addition, the taxa present in CRC were different depending on the CRC stage [66,67]. As happened with other omic layers, there have been differences detected in the microbiome composition between right-colon and left-colon cancer [69,70]. Thus, the microbiome signature could be used to predict different features of CRC.

## 3. Combination of the Analysis of Multiple Omic Layers

The combination of multiple omic layers allows the study of the interaction of the analyzed layers and the effect of that interaction on the trait, in this case, CRC. In this way, reliable and representative biomarkers can be inferred from each omic layer that can be useful to personalize the diagnostic or the treatment.

The analysis and integration of data from the genome (single-nucleotide variant and copy number variation), epigenome (DNA methylation), and transcriptome (mRNA) allowed the discovery of 12 genes that were useful for prognosis in I and II stages of CRC [71]. In addition, among those genes, the expression of *REP15* and *LRRC26* genes showed the best prognosis in the early stages of CRC [71]. In another study, the combination of the genome (copy number variation), transcriptome (gene and miRNA expression), and metabolome (from serum and urea) were used to find the molecular profiling of the relapse in I and II stages CRC [72]. First, each omic layer was analyzed to obtain the most informative features (16 copy number variations, 12 genes, 25 miRNAs, 24 serum metabolites, and 7 urine metabolites) and then those features were combined to obtain the most informative data and remove redundant information, gaining 31 informative features (2 copy number variations, 8 genes, 1 miRNA, 13 serum metabolites, and 7 urine metabolites) as predictors of relapse [72]. Thus, the use of different omic layers could be useful to find biomarkers for prognosis and, therefore, to adjust the treatment.

Moreover, another study used stemness (the capacity of self-renewal and repopulation of tumorous cells) to classify the tumors and link that classification to various omic layers [73]. First, the transcriptome was used to classify the tumors into low- and high-stemness groups, which have better and worse prognoses, respectively [73]. They concluded that nine genes (*GFPT1*, *PTMAP9*, *MOGAT3*, *DPM3*, *S100A12*, *PGM5*, *FUT6*, *SEMA3C*, and *ADAM33*) could be useful to perform the prediction of the groups, and they assessed their prognostic value [73]. The performance of classifying those genes was high in the five datasets they used for the validation [73]. Finally, the genomic layer (tumor mutation burden, SNPs, insertions and deletions, genome alterations, and copy number variations) was used to characterize the groups [73]: The group with the better prognosis was mutation-driven and the group with the worst prognosis was copy number deletion-driven. Thus, as relevant features of tumors are investigated and incorporated, stratification will become more refined, and therefore, multi-layered analyses can be used to find useful new markers.

Moreover, in a study comparing short-term and long-term survival to CRC, the expression of mRNA and miRNA and the bacterial composition were analyzed altogether to measure their ability to predict survival. The microbiome was the best omic layer predicting the survival of CRC patients (AUC of 0.755), followed by mRNA (AUC of 0.702), while miRNA showed the worst performance (AUC of 0.546) [74]. In another study, genome, metabolome, and microbiome data were used to assess the risk factors for CRC and, when analyzed altogether, the contribution of each layer was different [75]: The microbiome affected the most variance, the metabolome showed a smaller effect, and the contribution of the genome to the variance was limited. In addition, the covariance between the different layers was limited, and the use of features from the three layers showed better performance to predict CRC risk than using only two layers [68,75]. However, some features from the metabolome and microbiome that were useful to predict CRC risk could be influenced by the genome [68,75]. Thus, a part of the risk captured by each layer could be independent and another part could be redundant, depending on the biological mechanism involved.

## 4. Difficulties and Future Steps

We have reviewed how to take advantage of omic layers towards personalized medicine and it is clear that, although remarkable progress has been made, we are not yet there. In this section, we will discuss the difficulties that we have to face and the possible steps to overcome them.

Although the data from various omics layers from CRC samples have been available for some time and it was suggested that this would serve to improve knowledge about CRC [49], it seems that their use in clinical practice has not been as general as we would expect. For example, the search for non-invasive biomarkers has led to the finding of biomarkers from the blood that seems promising [76]. Those biomarkers were from the epigenome layer (DNA methylation and microRNA) and their combination is necessary to reach an optimal performance [76]. In addition, the contribution, involvement, and collaboration of different stakeholders are needed for the success of the application of those biomarkers in screening programs [76].

**Figure 1:**
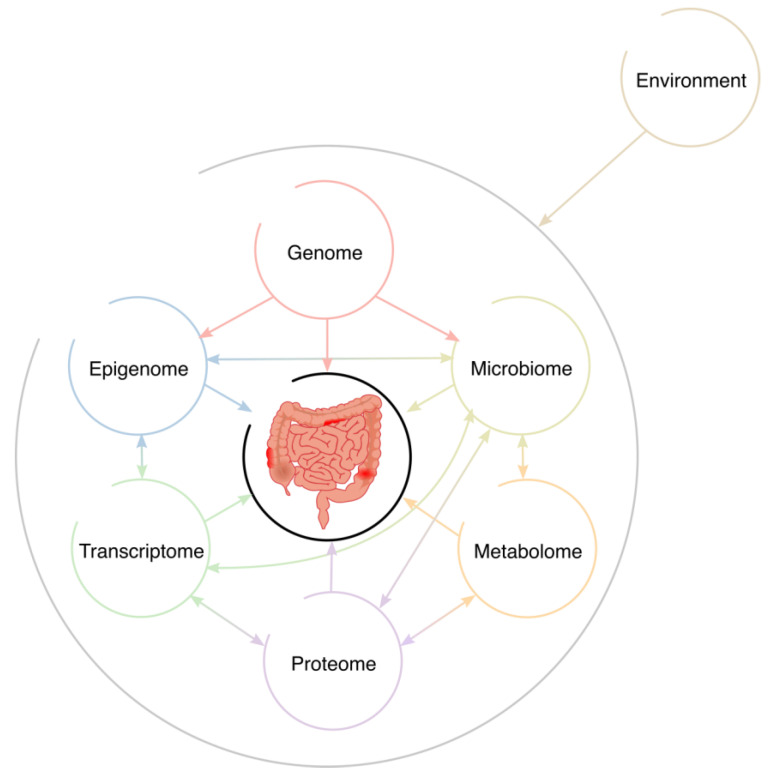
Interactions between omic layers and the environment that could lead to colorectal cancer development.

It has to be highlighted that the effect of each omic layer can be inherited from the previous omic layer (e.g., the effect of a given transcript can be a consequence of a mutation of the genome), the effect can emerge in that omic layer, and the effect can be in a different direction (Figure 1). In other words, the phenotype could be a consequence of a linear variation of the omic layer, or it could be the combination of the variation of each omic layer [77]. Thus, depending on the omic layers analyzed and the biological mechanisms involved, the biomarkers proposed may not accurately represent all the variation, and their performance would not be optimal. Previously, we have discussed that some markers for each layer can be redundant [72] and others complementary [68,75], since part of the risk to CRC or its outcomes seems to be inherited from a previous omic layer and another part of the risk seems to emerge in one omic layer [68,75]. Thus, one must evaluate the contribution of each omic layer and each marker to avoid overlaps or losing information.

Although the technologies to sequence and analyze omic layers have been improved, we still have several challenges when incorporating omic information into personalized medicine [78]: The cost of those technologies is not affordable for everyone, and the storage, process, integration, and interpretation of the data could be a limitation. These issues are of concern in some omic layers. For example, in microbiome analyses, the conciliation of different studies is difficult, not only because of the biological variability between populations but also due to methodological variability [65]. In addition, the validation of the markers in different populations is not as widespread as would be expected, maybe because it is not affordable or possible to analyze all the relevant omic features in the samples that are available.

Moreover, we cannot forget the effect of the environment [8] and socioeconomic status [79] on the risk and development of CRC (Figure 1). In addition, the use of clinical data can improve the prediction capability of omic markers [68,75]. The incorporation of these data will be necessary to improve omic analyses and avoid biases due to confounding factors that are not usually considered.

However, we have an exciting road ahead. The development of single-cell technologies, the improvement of computational capabilities, the availability of open methods and data, and the advancement of medical care and devices are an opportunity to overcome the limitations. In addition, we will need the incorporation of new populations to be possible for the validation of markers or to find markers that are relevant for those populations for predicting and personalizing CRC diagnosis, prognosis, and treatment.

## 5. Conclusions

The information from omic layers can be used to obtain biological markers that could improve and personalize the diagnosis, prognosis, and treatment of colorectal cancer. As more omic information has been incorporated, we have realized that the effect of each omic layer and its features could be redundant or complementary. In addition, as more populations have been analyzed, we have realized that the performance of the omic markers can be variable. Thus, the omic markers should be evaluated in terms of utility for a population and non-redundancy. We will need fresh ideas to extract the maximum information from each omic layer, integrate the omic layers, and validate the more feasible omic markers in a wide range of populations, to make personalized medicine useful and accessible to all CRC patients.

## Data Availability

Not applicable.
